# Epidemiology of bone tumors in Lebanon: a retrospective study from 2000 to 2022 at a tertiary center

**DOI:** 10.2144/fsoa-2023-0102

**Published:** 2023-08-08

**Authors:** Mohammad Daher, Ziad Zalaquett, Elio Mekhael, Marven Aoun, Ralph Chalhoub, Majd Abdo, Marc Aoude, Sami Abi Farraj, Ali Ghoul, Jean Tarchichi, Joe Rassi, Amer Sebaaly, Viviane Trak-Smayra, Carole Kesrouani, Hampig-Raphaël Kourie, Ismat Ghanem

**Affiliations:** 1 Hôtel Dieu de France, Orthopedic Department, Beirut, Lebanon; 2 Hôtel Dieu de France, Hematology-Oncology Department, Beirut, Lebanon; 3 Hôtel Dieu de France, Pathology Department, Beirut, Lebanon

**Keywords:** bone metastasis, bone tumors, epidemiology, Ewing's sarcoma, Middle-East, osteosarcoma

## Abstract

**Aim:**

Bone tumors are rare and have an uneven geographic distribution.

**Methods:**

730 patients diagnosed with bone tumors were included in this retrospective analysis.

**Results:**

With a 64% rate of malignancy, the most common tumors were metastasis (40%) mostly in the axial skeleton, Osteosarcoma (9%) mostly in the femur, Osteochondroma (8%) mostly in the femur, giant cell tumors (7%) mostly in the knee, and Ewing's sarcoma (6%) mostly in the axial skeleton.

**Conclusion:**

Even though a some of the tumors have a predilection for certain localizations in the human body, they may differ in the middle-eastern population. One must also pay attention to the higher rates of malignancies as compared with other cohorts.

## Plain language summary

With significant morbidity and mortality, bone tumors incidence is low and varies geographically. In our Lebanese population, Seven-hundred-thirty patients with bone tumors were identified with a 64% rate of malignancy with osteosarcoma being the most common primary bone cancer and metastasis being the overall most prevalent bone malignancy. This higher rate of malignancy compared with other populations should be taken into consideration when evaluating Lebanese or Middle eastern patients.

Musculoskeletal tumors account for only 0.3% of all cancers at any age with lesions that simulate primary bone tumors being far more common (https://pubmed.ncbi.nlm.nih.gov/25703268/). They are most frequently observed in children and adolescents, with osteosarcoma and Ewing sarcoma (the most common primary bone tumors) occurring mostly between 10 and 20 years of age (https://link.springer.com/chapter/10.1007/978-3-540-77984-1_1). These tumors have a very uneven geographic distribution around the world, with the majority of cases documented in Western Europe and the USA [1]. In fact, bone tumors can be divided into numerous different subtypes based on the tissue that they create, such as vascular channels in angiosarcoma of the bone, or the direct result of the tumor cells, such as osteoid in osteosarcomas [2].

Despite their rarity, these tumors are a significant contributor to morbidity and mortality, especially those of malignant nature [3]. Therefore, advancing knowledge of their epidemiology, diverse types of presentation, and groups at risk are crucial. There is a dearth of precise data on the epidemiology of musculoskeletal tumors in the Middle East, particularly in Lebanon. Thus, the goal of this study was to determine the incidence, age and sex distribution, histologic type, and location of benign and malignant bone tumors, as well as tumor-like lesions of the bone at Hôtel-Dieu de France, a tertiary referral hospital in Beirut, Lebanon.

## Materials & methods

### Patients

Seven hundred thirty patients diagnosed with bone tumors aged from 0 to 94 years were included in this retrospective analysis from the department of orthopedics at Hôtel-Dieu de France Hospital, Beirut, Lebanon between January 2000 and December 2022. Every patient diagnosed with bone tumors was included in this study. The protocol was approved by the local ethics committee at both Saint Joseph University and Hôtel-Dieu de France Hospital which serves a big part of Beirut the capital, as well as receiving cases from all over Lebanon. For each patient, the data which was collected (and was the only data available) prospectively by the different orthopedic surgeons and pathology department included the age of presentation, gender, diagnosis (which was made using biopsy or imaging), and localization.

### Statistical analysis

In this study, the normality of age was assessed using the Shapiro–Wilk test. To compare the proportions of genders across tumor groups, a Chi-square test was employed. To compare the age at diagnosis between gender groups in the whole cohort and within tumor groups, a Mann–Whitney test was employed. The statistical analysis was carried out using SPSS^®^ (version 26, IBM, USA), and a probability value of less than 0.05 was considered statistically significant.

## Results

**Figure 1.**
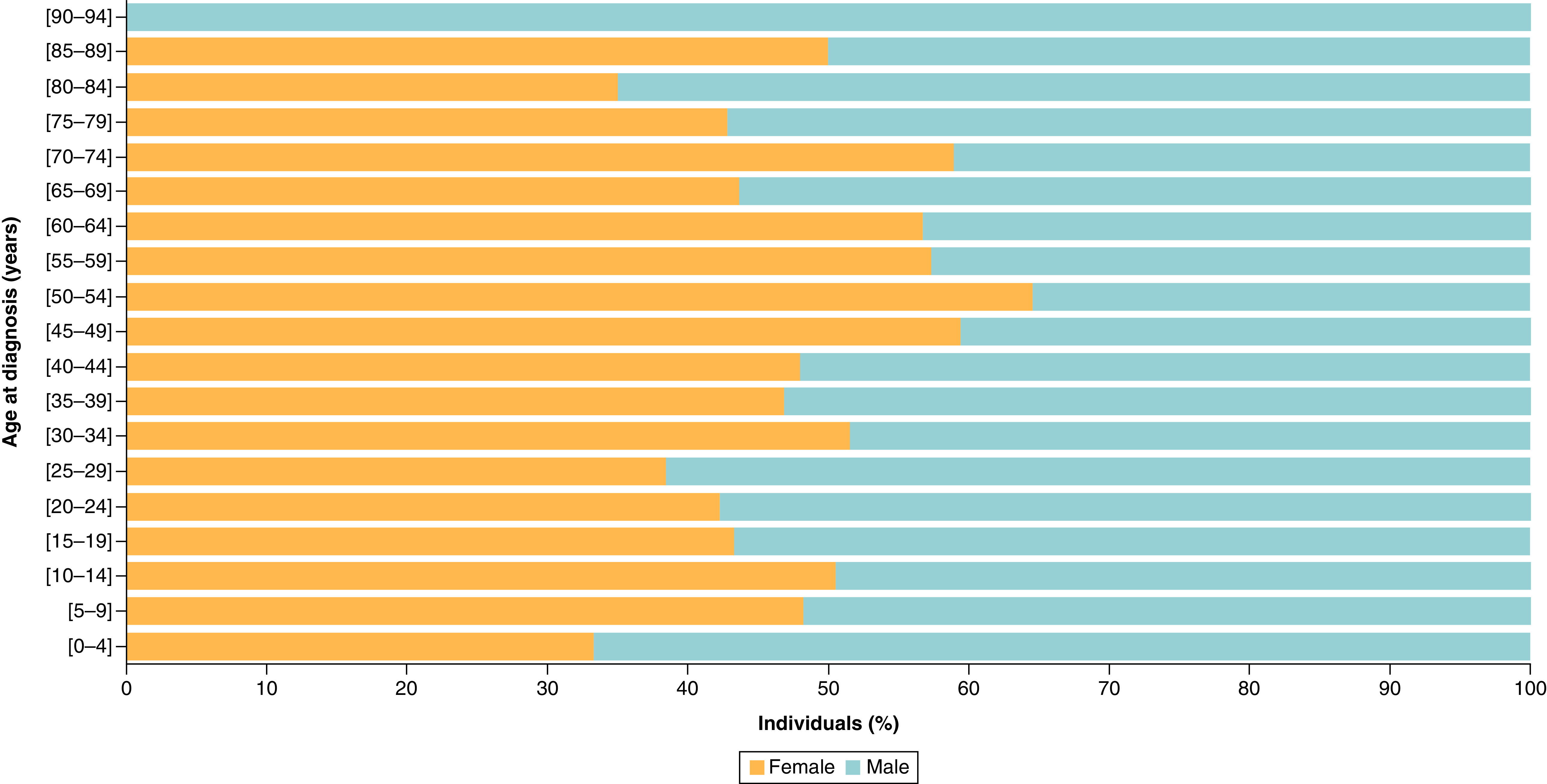
Age pyramid showing the different age distributions across males and females.

The Shapiro–Wilk test revealed that patients' age is normally distributed (p > 0.05). Our population consisted of 730 patients (age: 40.7 ± 24.4 years) in total diagnosed with different tumor types. Benign tumors constituted 36% of this cohort, with 64% being malignant tumors. Chi-square test revealed that there were no difference in gender across tumor groups (p > 0.05). The Mann–Whitney test revealed that the age at diagnosis was the same between the 363 females (age: 41.6 ± 23.6 years) and the 367 males (age: 39.9 ± 25.2 years) (p = 0.44, Figure 1 & Table 1).

**Table 1.** Table showing gender distribution across the age at diagnosis.

Years	Female	Male
0–4	6	12
5–9	28	30
10–14	47	46
15–19	26	34
20–24	19	26
25–29	10	16
30–34	17	16
35–39	15	17
40–44	12	13
45–49	19	13
50–54	31	17
55–59	39	29
60–64	38	29
65–69	24	31
70–74	23	16
75–79	15	20
80–84	7	13
85–89	1	1
90–94	0	4
Total	377	383

In order to classify all of the tumor types, 6 groups were formed based on the number of patients presenting each tumor type: the 5 tumor type groups with the biggest number of patients formed the following 5 groups: “Bone metastasis”, “Osteosarcoma”, “Osteochondroma”, “Giant Cell Tumors” and “Ewing's sarcoma”. The rest of the tumor type groups with fewer number of patients formed the last group which will be referred to as “Others”. Localizations for each of the latter 5 tumor type groups were presented in Table 2 with the number of individuals in each group.

**Table 2.** Table showing tumor localization distribution across tumor groups.

Localization	Tumor type					Total
	Ewing's sarcoma	Giant cell tumor	Metastasis	Osteochondroma	Osteosarcoma	
Ankle	2	2	0	3	3	10
Collarbone	0	0	5	0	0	5
Elbow	0	2	0	1	0	3
Femur	7	3	42	11	32	95
Fibula	3	1	0	1	1	6
Foot	1	4	0	4	1	10
Forearm	1	0	0	1	0	2
Hand	0	5	0	3	0	8
Hip	0	0	17	2	0	19
Humerus	2	0	21	6	3	32
Knee	0	9	2	9	4	24
Lumbar	0	0	38	0	0	38
Lumbo-sacral	2	1	0	0	0	3
Pelvis	6	2	31	0	2	41
Radius	0	2	0	0	0	2
Ribs, vertebra, paravertebral	8	6	106	2	4	126
Sacrum	0	0	18	0	0	18
Scapula	1	0	5	2	1	9
Shoulder	2	2	2	1	1	8
Skull	0	0	1	0	0	1
Sternum	0	0	2	0	0	2
Tibia	8	7	2	10	17	44
Ulna	0	1	1	0	0	2
Wrist	0	1	0	0	0	1
Total	43	48	293	56	69	509

### Bone metastasis

Out of the 730 patients, 293 were diagnosed with bone metastasis (40%). Mean age in this group was 60.1 ± 14.6 years, median age was 61 years with a maximum of 94 and a minimum of 0 years, with 55% (n = 161) being female patients (mean age: 59.7 ± 11.9 years, median: 60 years, maximum: 86 years, minimum: 15 years) and 45% (n = 132) being male patients (age: 60.6 ± 17.5 years, median: 63 years, maximum: 94 years, minimum: 0 years), with no difference in age at diagnosis (p > 0.05). The most common localization among patients with bone metastasis was the ribs, vertebrae and paravertebral (36%) (Table 3).

**Table 3.** Table showing the number of patients with bone tumor cases and characteristics in the Hotel Dieu de France database for 2000–2022.

Type	n (%)	Localization	Mean age (Years)	Standard deviation	Sex	n (%)	Mean age (years)	Standard deviation
Metastasis	293 (40%)	Ribs, Vertebra, Paravertebral (n = 106)	60.1	14.6	F	161 (55%)	59.7	11.9
					M	132 (45%)	60.6	17.5
Osteosarcoma	69 (9%)	Femur (n = 32)	26.0	19.4	F	33 (48%)	29.0	22.9
					M	36 (52%)	23.3	15.4
Osteochondroma	56 (8%)	Femur (n = 11)	22.5	17.6	F	24 (43%)	19.1	13.3
					M	32 (57%)	25.0	20.0
Giant Cell Tumor	48 (7%)	Knee (n = 9)	29.3	18.9	F	26 (54%)	28.2	15.9
					M	22 (46%)	30.5	22.3
Ewing's Sarcoma	43 (6%)	Ribs, Vertebra, Paravertebral (n = 8)	20.6	14.1	F	15 (35%)	17.7	8.4
					M	28 (65%)	22.1	16.2
Total	509 (70%)	Ribs, Vertebra, Paravertebral (n = 126)	45.1	23.8	F	259 (51%)	46.4	22.3
					M	250 (49%)	43.7	25.3

### Osteosarcoma

Out of the 730 patients, 69 were diagnosed with osteosarcoma (9%). Mean age in this group was 26 ± 19.4 years, median age was 17 years with a maximum of 81 and a minimum of 6 years, with 48% (n = 33) being female patients (mean age: 29 ± 22.9 years, median: 15 years, maximum: 81 years, minimum: 9 years) and 52% (n = 36 being male patients, mean age: 23.3 ± 15.4 years, median: 18.5 years, maximum: 69 years, minimum: 6 years), with no difference in age at diagnosis (p > 0.05). The most common localization among patients with osteosarcomas was the femur (46%) (Table 3).

### Osteochondroma

Out of the 730 patients, 56 were diagnosed with osteochondroma (8%). Mean age in this group was 22.5 ± 17.6 years, median age was 16 years with a maximum of 77 and a minimum of 1 year, with 43% (n = 24) being female patients (mean age: 19.1 ± 13.3 years, median: 14.5 years, maximum: 56 years, minimum: 7 years) and 57% (n = 32) being male patients (mean age: 25 ± 20 years, median: 16.5 years, maximum: 77 years, minimum: 1 year), with no difference in age at diagnosis (p > 0.05). The most common localization among patients with osteochondroma was the femur (20%) (Table 3).

### Giant cell tumors

Out of the 730 patients, 48 were diagnosed with giant cell tumors (7%). Mean age in this group was 29.3 ± 18.9 years, median age was 23 years with a maximum of 91 and a minimum of 5 years, with 54% (n = 26) being female patients (mean age: 28.2 ± 15.9 years, median: 23.5 years, maximum: 68 years, minimum: 8 years) and 46% (n = 22) being male patients (mean age: 30.5 ± 22.3 years, median: 22 years, maximum: 91 years, minimum: 5 years), with no difference in age at diagnosis (p > 0.05). The most common localization among patients with giant cell tumors was the knee (19%) (Table 3).

### Ewing's sarcoma

Out of the 730 patients, 43 were diagnosed with Ewing's sarcoma (6%). Mean age in this group was 20.6 ± 14.1 years, median age was 18 years with a maximum of 82 and a minimum of 2 years, with 35% (n = 15) being female patients (mean age: 17.7 ± 8.4 years, median: 16 years, maximum: 33 years, minimum: 4 years) and 65% (n = 28) being male patients (mean age: 22.1 ± 16.2 years, median: 19 years, maximum: 82 years, minimum: 2 years), with no difference in age at diagnosis (p > 0.05). The most common localization among patients with Ewing's sarcoma was the ribs, vertebrae and paravertebral (19%) (Table 3).

### Others

Out of all the tumor types forming the “Others” group (including tumor-like lesions of the bone), osteoid osteoma was the most prevalent, being present in 31 patients. The rest of the findings in this group are summarized in Table 4.

**Table 4.** Table showing the different tumors in the “Others” group (including tumor-like lesions of the bone).

Type	n (%)	Type	n (%)
Osteoid osteoma	31 (4.2)	Fibrosarcoma	3 (0.4)
Aneurysmal bone cyst	27 (3.7)	Angiosarcoma	3 (0.4)
Chondrosarcoma	23 (3.2)	Myosarcoma	3 (0.4)
Lipoma	18 (2.5)	Angiolipoma	2 (0.3)
Hemangioma	16 (2.2)	Chondroma	2 (0.3)
Enchondroma	14 (1.9)	Cystic tumor	2 (0.3)
Liposarcoma	11 (1.5)	Other bone cyst	2 (0.3)
Neuroma	10 (1.4)	Angioma	1 (0.1)
Histiocytoma	9 (1.2)	Chondromyxoid fibroma	1 (0.1)
Langerhans cell histiocytosis	9 (1.2)	Elastofibroma	1 (0.1)
Sarcoma	9 (1.2)	Fibrous histiocytoma	1 (0.1)
Fibroma	7 (1)	Lytic Tumor	1 (0.1)
Carcinoma	6 (0.8)	Myofibroma	1 (0.1)
Osteoblastoma	6 (0.8)	Myxoma	1 (0.1)
Schwannoma	6 (0.8)	Neorectum	1 (0.1)
Synovial Cyst	5 (0.7)	Non specified inflammatory modification	1 (0.1)
Lymphangioma	4 (0.6)		
Plasmocytoma	4 (0.6)	Round cell tumor – unclassified	1 (0.1)
Multiple myeloma	4 (0.6)	T lymphocyte leukemia	1 (0.1)
Chondroblastoma	3 (0.4)	Undifferentiated	1 (0.1)

## Discussion

Primary bone tumors are uncommon, and their clinical signs are frequently ambiguous. Patients may exhibit pain, edema, or both, or in up to 35% of instances, a pathologic fracture may be the presenting symptom without really supporting a particular diagnosis [4]. In most series published globally [5–7], primary bone tumors account for 0.2% to 0.5% of all malignancies [6,8]. A review of the international literature reveals that benign bone tumors are much more common than malignant ones [3,9–12]. However, this was not seen in our patients, where malignant tumors made up 64% of our cohort. Nevertheless, if bone metastasis were excluded from our cohort, malignant tumors would still make up 40%, which would still be higher than most studies reporting an incidence of around 20% [3,9–12]. While the high incidence of malignant tumors could be related to the increased referral of malignant cases to our hospital, we highly doubt it is fully responsible for the large difference in incidence as compared with benign tumors. Moreover, a study by Ozturk *et al.* [13] showed an incidence of malignant tumors around 48% (even though the incidence of bone metastasis was low) in a Turkish population, which is a country in the Middle East close to Lebanon. This raises the question of the implication of a geographical factor that may be related to this high malignancy incidence. The incidence of these different malignant tumors varied as well which could be explained by other factors such as genetic predisposition to certain tumor types, or simply referral patterns, if certain types are more commonly referred to our hospital. Future studies should aim to investigate these factors in more detail to better understand the underlying reasons for the observed differences in tumor incidence. Furthermore, it is important to highlight that young patients in their second decade of life had the highest incidence of both benign and malignant bone tumors in our analysis, a finding that was also found in another study [3]. This may insinuate that among the general population, young people had the highest risk of developing malignant tumors.

Our study presented 730 cases over a 22-year period. Bone metastases were shown to be the most common (40%). In other cohorts, these tumors were the second-most frequent type of bone malignancy, with an incidence of 18.6% [3]. Bergovec *et al.*, however, reported bone metastasis to be the third-most common malignant tumor [12]. The most common site of these malignancies was the axial skeleton, which was similar to other studies [3,14]. In our cohort, bone metastases were more frequent in females than males, with an incidence of 55% and 45%, respectively. This may be due to the high contribution of breast cancer to bone metastasis. However, no difference in the age of occurrence, which was around 60 years, was recorded between the two sexes.

Osteosarcoma represents the most common primary malignant bone neoplasm [10,13,15–17] with an incidence ranging between 35.1% and 47% of bone tumors [3,6]. This was further demonstrated in our cohort, as osteosarcoma was found to be the most common primary bone malignancy. Its incidence of 9%, however, is relatively low compared with other cohorts. The age predominance of this tumor was around 26 years, with no difference between males and females. However, it was shown to occur mostly between 11 and 20 years of age in another study [3]. This age discrepancy could therefore be due to a geographical difference in the populations, or simply because of late diagnosis in our country. Osteosarcoma was shown to have a predilection for the femur, with 46% occurring in this location, a finding that can be confirmed by other studies as well [3,18].

In our cohort, Ewing sarcoma was seen to be the fifth-most common primary bone tumor and third-most common bone malignancy accounting for 6% of all tumors. This incidence varies between studies, being 2.5% in a study by Baena-Ocampo *et al.* [3] and 8.6% in the Mayo Clinic series [18,19]. Furthermore, Unni *et al.* showed that this malignancy is the fourth-most common primary malignant neoplasm of the bone [18]. Ewing sarcoma mostly affected men (65%), which was also seen in other studies [7,8,20]. The mean age was around 20.6 years, slightly higher than what is reported in the literature as being between 10 and 20 years [21]. As for the localization, a difference was seen between our patients where this malignancy was most frequently seen in the axial skeleton and the other cohorts where the most common localization was the tibia [3,22].

Chondrosarcoma was shown to be the second-most common malignant tumor in most of the cohorts around the world [3,6,12,18], and sometimes the first [23]. However, in our cohort it was the fourth-most frequent malignant bone tumor, and the eighth-most common primary bone tumor (benign and malignant), accounting for only 3% of all tumors. This finding was most similarly seen in a study by Ozturk *et al.* [13] where a lower incidence of chondrosarcoma occurred in a Turkish cohort and was ranked as the third-most common malignant tumor.

As for benign tumors, the third-most common bone tumor was osteochondroma, and it was the most common benign bone tumor, accounting for 8% of all bone tumors. Although it has been seen as the most common benign bone tumor in other studies [3,6,10,12,13,16,17,20], its incidence was much higher, at 28.5% in a study conducted in a Mexican population [3], and 21.3% in a Croatian population [12]. However, Unni *et al.* reported a 9.9% incidence, which is more similar to our cohort [18,19]. This benign tumor occurred mainly in males (57%), a finding seen in other cohorts as well [24,25]. A difference was noted in its localization : it was mostly the femur in our patients (20%), whereas it is mostly the shin bones in the literature [24,26]. Giant cell tumor of bone was reported to represent 5% of all bone tumors in the literature [27]. This incidence rate is similar to ours, which was 6%, making this tumor the fourth-most common tumor in our cohort, and the second-most common benign tumor overall. Baena-Ocampo *et al.* [3] reported this tumor to be the second-most commonly seen benign bone tumor as well. However, in the Mayo Clinic series, the incidence was around 21.9% [18,19]. The age at incidence was around 29.3 years, which is slightly lower than what is reported in the literature, which is a range of 30.5 to 35.7 years [28,29]. Furthermore, we showed a higher prevalence in female patients, whereas Liede *et al.* reported a male predominance [30].

### Strengths & limitations

Although thorough, this report does not necessarily depict with accuracy the incidence of all musculoskeletal tumors in our country; rather, it depicts the incidence of those that were identified and/or treated in our department over a 22-year period. Nevertheless, the incidence of musculoskeletal tumors presented in this report is believed to be very accurate and provides a high-quality picture of their epidemiology in our country.

## Conclusion

This study provides an overview of trends in musculoskeletal tumors over a 22-year period in a consistent Lebanese population, with information that may be relevant to several nearby nations. The anatomical distribution of tumors presented here further affirms that these cancers have a persistent preference for particular localizations, and knowledge of the afflicted anatomical location can facilitate a faster and more accurate diagnosis. Although studies addressing these issues have very different geographical and sociological backgrounds, one can clearly see recurring patterns in their findings. Nevertheless, when considering Middle Eastern population, one must consider the higher rate of bone metastasis, which occurred mainly in females, the lower rate of chondrosarcomas, and the different localization of some of these malignancies.

**Summary points**

- A higher rate of malignancy was seen in this Lebanese cohort.
- The most common tumors included: metastasis, osteosarcoma, osteochondroma, giant cell tumor, Ewing's sarcoma.
- The patients with the highest incidence of bone tumors were in their second decade.
- Metastasis were mostly seen in females.
- Different localizations of these tumors were seen as well.
- A lower rate of chondrosarcoma was recorded.
